# Association between periodontitis and cardiovascular disease: a retrospective analysis

**DOI:** 10.1186/s12903-026-08282-x

**Published:** 2026-04-06

**Authors:** Yi Lu, Baojun Lu

**Affiliations:** Department of Stomatology, Baoying Traditional Chinese Medicine Hospital, No.58 Taishan West Road, Baoying County, Yangzhou, Jiangsu Province 225800 China

**Keywords:** Periodontitis, Cardiovascular disease, Retrospective study, Risk factors, Oral-systemic health

## Abstract

**Background:**

Periodontitis is a common chronic inflammatory disease that has been increasingly linked to systemic conditions, including cardiovascular disease (CVD). The inflammatory burden and bacterial dissemination associated with periodontitis may contribute to atherosclerosis and cardiovascular events. However, evidence from clinical retrospective cohorts in Chinese populations remains limited.

**Methods:**

This retrospective observational study was conducted at the Baoying Traditional Chinese Medicine Hospital between January 2021 and December 2022. A total of 472 adult patients were included. Clinical and demographic data, periodontal parameters, and cardiovascular histories were collected from hospital records. Patients were classified according to the presence or absence of periodontitis. The prevalence of cardiovascular diseases—including coronary artery disease, myocardial infarction, and stroke—was compared between groups. Multivariate logistic regression and stratified subgroup analyses were performed to identify independent associations between periodontitis and cardiovascular outcomes, adjusting for potential confounders such as age, sex, smoking status, diabetes, and body mass index.

**Results:**

Among 472 patients, 289 (61.2%) had clinically confirmed periodontitis, while 183 (38.8%) did not. The prevalence of cardiovascular disease was significantly higher in the periodontitis group compared with controls (38.7% vs. 21.3%, *P* < 0.001). Multivariate logistic regression revealed that periodontitis was independently associated with increased risk of cardiovascular disease (adjusted OR = 1.87, 95% CI: 1.28–2.72, *P* = 0.001), even after adjustment for traditional risk factors. Additional predictors included age ≥ 60 years, smoking history, and diabetes. Additional severity-stratified analyses suggested that the association with cardiovascular disease was stronger in patients with stage III/IV and grade C periodontitis than in those with milder disease.

**Conclusion:**

This retrospective study found a significant association between periodontitis and cardiovascular disease after adjustment for conventional risk factors. These findings support the relevance of periodontal health in comprehensive cardiovascular risk assessment, although prospective studies are needed to clarify causality and the potential impact of periodontal treatment on cardiovascular outcomes.

**Supplementary Information:**

The online version contains supplementary material available at 10.1186/s12903-026-08282-x.

## Introduction

Cardiovascular disease (CVD) remains the leading cause of morbidity and mortality worldwide, imposing a substantial burden on healthcare systems [1–3]. Traditional risk factors such as age, hypertension, diabetes, smoking, and obesity are well established; however, increasing evidence suggests that chronic inflammatory diseases may also play a key role in cardiovascular pathogenesis. Among these, periodontitis—a chronic dysbiotic inflammatory disease of the tooth-supporting tissues—has gained attention as a potential contributor to cardiovascular risk [4–6]. 

Periodontitis is now understood as a complex dysbiotic condition characterized by an imbalance in the subgingival microbiota, which triggers a cascade of immune responses. This includes the overproduction of inflammatory mediators and immune dysregulation, such as impaired phagocytosis and diminished responses from T lymphocyte subsets, particularly Tregs, leading to a chronic inflammatory state [7–9]. These inflammatory processes may accelerate endothelial dysfunction, promote atherosclerotic plaque formation, and increase the likelihood of thromboembolic events through sustained systemic inflammation and immune dysregulation [10–12]. Multiple epidemiological studies have reported a higher incidence of coronary artery disease, myocardial infarction, and stroke among patients with periodontitis, supporting a close association between oral inflammatory burden and CVD [6, 13, 14]. However, the strength of this association has varied across populations, likely due to heterogeneity in study design, periodontal diagnostic criteria, cardiovascular endpoints, and the extent of adjustment for traditional cardiovascular risk factors.

The relationship between periodontitis and CVD has been extensively studied, and many scientific heart societies, including the European Society of Cardiology, now recognize periodontitis as an independent associated factor for CVD. Numerous studies have shown a significant association, and while the exact mechanisms remain under investigation, the role of systemic inflammation and immune dysregulation is thought to contribute to this relationship [15, 16]. The systemic inflammatory burden caused by untreated periodontitis may exacerbate underlying cardiovascular conditions, yet this association is not routinely considered during patient assessment. Therefore, integrating oral health evaluation into cardiovascular disease prevention strategies may provide an opportunity to improve outcomes through holistic management.

Despite the growing recognition of the oral-systemic link, few retrospective studies in regional healthcare settings have specifically examined the association between periodontitis and cardiovascular disease within Chinese populations. Although numerous studies have demonstrated an association between periodontitis and cardiovascular disease, fewer investigations have specifically examined whether periodontitis remains independently associated with cardiovascular disease after comprehensive adjustment for conventional risk factors, particularly in Asian populations [17–19]. Notably, recent consensus reports from the European Federation of Periodontology (EFP) and the European Society of Cardiology recognize periodontitis as a cardiovascular risk factor, highlighting the clinical relevance of this association [6]. Accordingly, the aim of this retrospective study was to investigate the association between periodontitis and cardiovascular disease in a cohort of 472 patients over a two-year period. By evaluating clinical and demographic characteristics alongside cardiovascular outcomes, we sought to determine whether periodontitis was independently associated with cardiovascular disease after adjustment for conventional cardiovascular risk factors in a regional Chinese clinical cohort.

## Materials and methods

### Study design and patient selection

This retrospective observational study was conducted at Baoying Traditional Chinese Medicine Hospital, located in Baoying County, Yangzhou City, Jiangsu Province, China, between January 2021 and December 2022. The study adhered to the STROBE (Strengthening the Reporting of Observational Studies in Epidemiology) guidelines to ensure transparent and standardized reporting.

All eligible patients were consecutively enrolled during the study period to minimize selection bias. A total of 472 patients with complete medical and dental records were included. Eligible participants were adults aged ≥ 18 years who had undergone periodontal evaluation during routine or diagnostic visits and had documented cardiovascular health data in the hospital’s electronic medical records. Patients with incomplete clinical data, a history of malignant tumors, systemic inflammatory or autoimmune diseases (e.g., rheumatoid arthritis, lupus), or prior periodontal or cardiac surgery were excluded. These criteria were designed to minimize potential confounding while reflecting real-world clinical conditions. Although this was a retrospective study, a post hoc power analysis was performed to confirm adequacy of the sample size. The current cohort (*n* = 472) provided > 80% power to detect an odds ratio ≥ 1.5 for the association between periodontitis and cardiovascular disease, at a two-tailed α = 0.05 significance level.

### Data collection

Demographic and clinical data were extracted independently by two blinded investigators using the hospital’s information system. Collected variables included age, sex, body mass index (BMI), smoking and alcohol consumption, and comorbidities such as diabetes mellitus, hypertension, and chronic kidney disease. Smoking and drinking behavior were defined using standardized criteria: Current smoker: ≥1 cigarette/day for ≥ 6 months prior to enrollment; Former smoker: cessation ≥ 12 months before enrollment; Never smoker: lifetime consumption < 100 cigarettes; Participants who reported smoking for less than 6 months prior to enrollment were classified as non-current smokers. Current drinker: alcohol consumption ≥ 1 time per week for ≥ 6 months prior to enrollment. To minimize information bias, data entries were cross-verified, and discrepancies were resolved by consensus. Cardiovascular diagnoses were confirmed by cardiologists and retrieved from the hospital’s coded records, including coronary artery disease, myocardial infarction, heart failure, stroke, and hypertension. The exact temporal sequence between periodontal examination and the initial diagnosis of cardiovascular disease could not be consistently determined for all participants.

### Tools, indices, and reliability

Clinical periodontal examinations were performed using a UNC-15 manual periodontal probe (Hu-Friedy, Chicago, IL, USA). Probing depth (PD) and clinical attachment level (CAL) were measured at six sites per tooth (mesiobuccal, midbuccal, distobuccal, mesiolingual, midlingual, and distolingual).

Bleeding on Probing (BOP) was assessed per 10% increase according to the Ainamo and Bay criteria and expressed as the percentage of bleeding sites among total sites examined [20]. Oral hygiene status was evaluated using the Silness and Löe Plaque Index (PI) [21].

Radiographic assessment of alveolar bone loss was performed using digital periapical and bitewing radiographs (Planmeca ProX, Helsinki, Finland).

All examinations were conducted by two calibrated periodontists (Dr. Y.L. and Dr. BJ.L.). Calibration was performed on 20 non-study patients prior to data collection, achieving intra- and inter-examiner reliability coefficients (ICC = 0.85 for PD and ICC = 0.88 for CAL). These procedures ensured consistency and reproducibility across all assessments.

### Definition of variables

Periodontitis was defined and staged according to the 2017 World Workshop on the Classification of Periodontal and Peri-Implant Diseases and Conditions, jointly developed by the American Academy of Periodontology (AAP) and the European Federation of Periodontology (EFP) [22]. Patients were classified into Stages I–IV based on CAL, PPD, radiographic bone loss, and tooth loss attributable to periodontitis, and graded (A–C) according to disease progression and risk factors. Cardiovascular disease (CVD) was defined as a composite outcome including coronary artery disease, myocardial infarction, stroke, and chronic heart failure. This composite definition was adopted to capture overall cardiovascular burden and to ensure adequate statistical power in this retrospective cohort. Disease-specific outcomes were additionally analyzed to explore potential heterogeneity across different cardiovascular conditions. Hypertension was included as a comorbidity and covariate in the regression analyses and was defined as systolic blood pressure ≥ 140 mmHg and/or diastolic blood pressure ≥ 90 mmHg, or current antihypertensive treatment. Diabetes mellitus was defined by prior physician diagnosis, fasting plasma glucose ≥ 7.0 mmol/L, HbA1c ≥ 6.5%, or the use of hypoglycemic therapy. Body mass index (BMI) was calculated as weight (kg) divided by height squared (m²) and categorized according to World Health Organization criteria for Asian populations. Although BMI does not distinguish between fat mass and lean mass, it remains a widely used and validated indicator of cardiovascular risk in population-based epidemiological studies. Alternative anthropometric measures, such as waist circumference or waist-to-hip ratio, were not available in the electronic medical records due to the retrospective design of this study.

### Bias control and quality assurance

To control for potential information and selection bias, all data were double-entered and validated by a senior investigator. Examiners performing periodontal assessments were blinded to patients’ cardiovascular data. Consecutive enrollment further minimized selection bias.

Quality assurance procedures included routine data audits, standardized training, and calibration prior to data collection. All protocols followed the STROBE guidelines for observational research.

### Statistical analysis

Data analysis was performed using SPSS version 25.0 (IBM Corp., Armonk, NY, USA). Continuous variables were tested for normality using the Kolmogorov–Smirnov test. Normally distributed variables were presented as mean ± standard deviation (SD) and compared using Student’s t test, while skewed variables were expressed as medians with interquartile ranges (IQR) and compared using the Mann–Whitney U test. Categorical variables were expressed as numbers and percentages and compared using the chi-square test or Fisher’s exact test where appropriate. Logistic regression models were constructed to evaluate the independent association between periodontitis and cardiovascular disease. Model 1 included only unadjusted associations; Model 2 adjusted for age and sex; Model 3 further adjusted for smoking, alcohol consumption, diabetes, hypertension, and BMI. Results were presented as odds ratios (OR) with 95% confidence intervals (CI). A two-tailed P value < 0.05 was considered statistically significant. Missing data were minimal (< 5% for all variables) and were handled using complete-case analysis. Logistic regression assumptions were assessed prior to modeling, including the absence of multicollinearity among covariates and the linearity of continuous variables in the logit. No violations of model assumptions were detected.

### Ethical considerations

This study was approved by the Institutional Ethics Committee of Baoying Traditional Chinese Medicine Hospital (Approval No. 202413579514), and conducted in accordance with the ethical principles outlined in the Declaration of Helsinki. Since this was a retrospective study based on anonymized medical records, the requirement for individual informed consent was waived. All patient information was de-identified to ensure confidentiality.

## Results

### Patient inclusion and exclusion

**Fig. 1:**
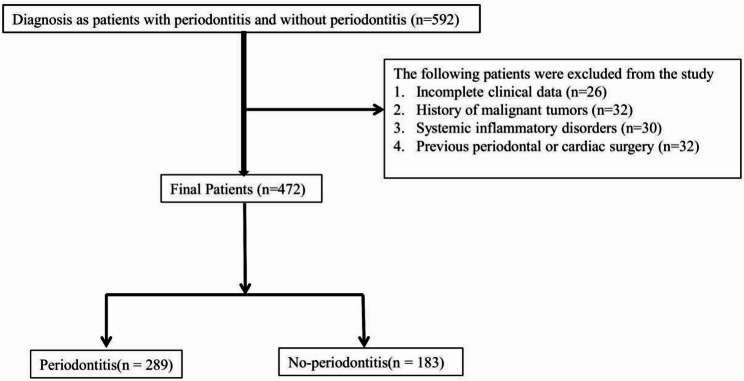
Flow diagram of patient screening, inclusion, and exclusion

Between January 2021 and December 2022, a total of 510 patients who had undergone both dental and cardiovascular evaluations were screened for eligibility. After excluding 18 patients with incomplete dental records, 12 patients with missing cardiovascular data, and 8 patients with systemic inflammatory or malignant diseases that could confound outcomes, 472 patients were finally included in the study. Of these, 289 (61.2%) were diagnosed with periodontitis, while 183 (38.8%) were classified as non-periodontitis. The process of inclusion and exclusion is detailed in Fig. 1, which illustrates the flow from initial screening to final enrollment.

### Baseline characteristics of patients

Baseline demographic and clinical characteristics are summarized in Table 1. The mean age of the study cohort was 58.8 ± 11.2 years, with no significant difference between the periodontitis and non-periodontitis groups (*P* = 0.529). Male patients accounted for 59.2% of the total cohort. Smoking exposure showed a clear dose–response relationship with periodontitis, with a higher proportion of current heavy smokers (≥ 10 pack-years) in the periodontitis group than in the non-periodontitis group (18.3% vs. 7.1%, *P* < 0.001). Compared with patients without periodontitis, those with periodontitis were more likely to have a history of diabetes mellitus (28.0% vs. 18.0%, *P* = 0.020). Hypertension was observed in 24.6% of patients with periodontitis and 15.3% of those without (*P* = 0.034). Patients with periodontitis presented a significantly greater mean probing pocket depth (4.8 ± 0.9 mm) compared with non-periodontitis patients (2.7 ± 0.5 mm, *P* < 0.001), and a higher percentage of sites with PPD ≥ 4 mm (32.4% vs. 6.7%, *P* < 0.001). No significant differences were observed between the groups with respect to alcohol consumption, body mass index (BMI), or prevalence of chronic kidney disease. These findings suggest that periodontitis patients tended to carry a higher burden of systemic comorbidities traditionally associated with cardiovascular disease.

**Table 1:** Baseline demographic and clinical characteristics of patients with and without periodontitis

Variables	Category	Periodontitis (*n* = 289)	Non-periodontitis (*n* = 183)	*P*-value
Age, years	Mean ± SD	59.1 ± 11.4	58.4 ± 10.9	0.529
Gender	Male	173 (59.9%)	106 (57.9%)	0.683
	Female	116 (40.1%)	77 (42.1%)	
BMI (kg/m²)	Mean ± SD	24.1 ± 3.6	23.8 ± 3.4	0.447
Smoking history	Non-smoker	190 (65.7%)	142 (77.6%)	< 0.001
	Former smoker (< 10 pack-years)	46 (15.9%)	28 (15.3%)	
	Current heavy smoker (≥ 10 pack-years)	53 (18.3%)	13 (7.1%)	
Alcohol consumption	Yes	87 (30.1%)	47 (25.7%)	0.321
Hypertension	Yes	71 (24.6%)	28 (15.3%)	0.034
Diabetes mellitus	Yes	81 (28.0%)	33 (18.0%)	0.020
Dyslipidemia	Yes	68 (23.5%)	34 (18.6%)	0.231
Chronic kidney disease	Yes	22 (7.6%)	10 (5.5%)	0.404
Chronic liver disease	Yes	19 (6.6%)	9 (4.9%)	0.462
Respiratory disease	Yes	24 (8.3%)	12 (6.6%)	0.527
Family history of CVD	Yes	65 (22.5%)	35 (19.1%)	0.392
Education level	College or higher	104 (36.0%)	72 (39.3%)	0.476
Physical activity	Regular	112 (38.7%)	77 (42.1%)	0.492
LDL cholesterol (mmol/L)	Mean ± SD	3.12 ± 0.94	2.98 ± 0.87	0.129
HbA1c (%)	Mean ± SD	6.7 ± 1.1	6.4 ± 0.9	0.047
Mean probing pocket depth (PPD, mm)	Mean ± SD	4.8 ± 0.9	2.7 ± 0.5	< 0.001
Sites with PPD ≥ 4 mm	% of sites per patient (Mean ± SD)	32.4 ± 11.8	6.7 ± 3.2	< 0.001
Clinical attachment loss (CAL, mm)	Mean ± SD	4.2 ± 1.0	2.3 ± 0.7	< 0.001
Clinical attachment loss	≥ 3 mm	201 (69.6%)	—	—

*Abbreviations*: *BMI* body mass index, *CVD* cardiovascular disease, *LDL* low-density lipoprotein, *HbA1c* glycated hemoglobin

### Association between periodontitis and cardiovascular disease

When the prevalence of CVD was compared between the two groups, a clear disparity was noted. As shown in Table 2 and 38.7% of patients with periodontitis were diagnosed with CVD, compared to only 21.3% in the non-periodontitis group (*P* < 0.001). Coronary artery disease was the most common cardiovascular outcome, occurring in 21.1% of patients with periodontitis versus 12.0% of those without. Myocardial infarction was also more frequent among patients with periodontitis (8.7% vs. 3.8%, *P* = 0.041). Similarly, the prevalence of stroke was modestly higher in the periodontitis group (6.9% vs. 3.3%, *P* = 0.084), though this difference did not reach statistical significance. Taken together, these results indicate that periodontitis is associated with an increased cardiovascular burden across multiple disease categories. Among individual cardiovascular outcomes, the association between periodontitis and cardiovascular disease appeared to be primarily driven by atherosclerotic conditions, particularly coronary artery disease and myocardial infarction, whereas associations with stroke and heart failure were weaker and did not consistently reach statistical significance.

**Table 2:** Prevalence of cardiovascular outcomes in the periodontitis and non-periodontitis groups

Cardiovascular outcome	Periodontitis (*n* = 289)	Non-periodontitis (*n* = 183)	*P*-value
Any cardiovascular disease	112 (38.7%)	39 (21.3%)	< 0.001
Coronary artery disease	61 (21.1%)	22 (12.0%)	0.015
Myocardial infarction	25 (8.7%)	7 (3.8%)	0.041
Stroke	20 (6.9%)	6 (3.3%)	0.084
Heart failure	12 (4.2%)	5 (2.7%)	0.417

*Abbreviations*: *CVD* cardiovascular disease

### Univariate and multivariate logistic regression

To further clarify the relationship between periodontitis and cardiovascular disease, logistic regression analyses were performed (Table 3). In univariate analysis, periodontitis, age ≥ 60 years, smoking history, diabetes, and hypertension were all significantly associated with CVD. In the multivariate model, after adjusting for age, sex, BMI, smoking, diabetes, and hypertension, periodontitis remained independently associated with the presence of CVD after adjustment for conventional cardiovascular risk factors (adjusted OR = 1.87, 95% CI: 1.28–2.72, *P* = 0.001). Other significant predictors included older age (adjusted OR = 1.65, 95% CI: 1.14–2.39, *P* = 0.008), smoking (adjusted OR = 1.52, 95% CI: 1.02–2.25, *P* = 0.038), and diabetes (adjusted OR = 1.61, 95% CI: 1.05–2.48, *P* = 0.028). These results emphasize that, even when controlling for conventional cardiovascular risk factors, periodontitis remained independently associated with CVD. After multivariate adjustment, significant associations were observed between periodontal indicators and several cardiovascular outcomes. Each 1 mm increase in probing pocket depth (PPD) was associated with a 25% higher risk of coronary artery disease (adjusted OR = 1.25; 95% CI: 1.08–1.45; *P* = 0.003), while each 1 mm increase in clinical attachment loss (CAL) increased the odds of myocardial infarction by 29% (adjusted OR = 1.29; 95% CI: 1.10–1.51; *P* = 0.002). A 10% increase in bleeding on probing (BOP) was associated with an 11% higher odds of cardiovascular disease (adjusted OR = 1.11; 95% CI: 1.04–1.19; *P* = 0.001). Radiographic alveolar bone loss ≥ 30% was independently related to higher risks of both stroke (adjusted OR = 1.52; 95% CI: 1.10–2.09; *P* = 0.011) and heart failure (adjusted OR = 1.46; 95% CI: 1.03–2.08; *P* = 0.034). These results suggest that greater periodontal tissue destruction is associated with progressively higher cardiovascular risk.

**Table 3:** Univariate and multivariate logistic regression analysis of predictors of cardiovascular disease

Variables	Category	Univariate OR (95% CI)	*P*-value	Multivariate OR (95% CI)	*P*-value
Periodontitis	Yes vs. No	2.28 (1.52–3.43)	< 0.001	1.87 (1.28–2.72)	0.001
Age	≥ 60 vs. < 60	1.71 (1.21–2.42)	0.003	1.65 (1.14–2.39)	0.008
Gender	Male vs. Female	1.12 (0.79–1.58)	0.528	1.09 (0.75–1.58)	0.648
Smoking history	Yes vs. No	1.68 (1.16–2.42)	0.006	1.52 (1.02–2.25)	0.038
Alcohol consumption	Yes vs. No	1.21 (0.83–1.77)	0.319	1.17 (0.78–1.75)	0.446
Diabetes mellitus	Yes vs. No	1.78 (1.21–2.64)	0.003	1.61 (1.05–2.48)	0.028
Hypertension	Yes vs. No	2.14 (1.46–3.14)	< 0.001	1.84 (1.23–2.75)	0.003
Dyslipidemia	Yes vs. No	1.34 (0.90–2.00)	0.149	1.27 (0.84–1.92)	0.260
BMI	≥ 25 vs. < 25 kg/m²	1.29 (0.89–1.87)	0.173	1.22 (0.82–1.82)	0.319

*Abbreviations*: *OR* odds ratio, *CI* confidence interval, *BMI* body mass index

To further assess whether the association varied by periodontal severity, we performed a stratified analysis according to periodontal stage and grade. As shown in Supplementary Table 1, a severity-dependent gradient was observed: patients with stage III/IV periodontitis and grade C periodontitis had higher cardiovascular disease prevalence and stronger adjusted associations than those with stage I/II and grade A/B disease. Specifically, stage III/IV periodontitis remained independently associated with cardiovascular disease after multivariable adjustment (adjusted OR 2.41, 95% CI 1.48–3.91; *P* < 0.001), whereas the association for stage I/II disease was weaker and not statistically significant (adjusted OR 1.32, 95% CI 0.76–2.29; *P* = 0.317). Similarly, grade C periodontitis showed an independent association with cardiovascular disease (adjusted OR 2.28, 95% CI 1.37–3.79; *P* = 0.002), while the association for grade A/B disease was attenuated after adjustment (adjusted OR 1.49, 95% CI 0.89–2.49; *P* = 0.129).

### Subgroup analysis

Subgroup analyses were conducted to explore whether the association between periodontitis and cardiovascular disease differed across predefined strata, including age, sex, smoking status, and body mass index (BMI) (Table 4). The association between periodontitis and cardiovascular disease remained statistically significant across most subgroups, including men and women, smokers and non-smokers, and participants with BMI above or below 25 kg/m², suggesting overall consistency of the observed association. Although variations in the magnitude of effect estimates were observed across certain strata, most interaction tests were not statistically significant, indicating no strong statistical evidence of effect modification. Therefore, all subgroup analyses should be regarded as exploratory, and the observed differences across subgroups may reflect random variation or limited statistical power rather than true heterogeneity of effect.

**Table 4:** Subgroup analysis of the association between periodontitis and cardiovascular disease

Subgroup	Adjusted OR (95% CI)	*P*-value	Interaction *P*-value
Age < 60 years	2.05 (1.21–3.46)	0.007	0.128
Age ≥ 60 years	1.41 (0.95–2.09)	0.087	
Male	1.82 (1.17–2.82)	0.008	0.421
Female	1.94 (1.09–3.48)	0.024	
Non-smoker	1.73 (1.09–2.75)	0.021	0.335
Smoker	1.95 (1.08–3.50)	0.027	
BMI < 25 kg/m²	1.79 (1.17–2.74)	0.007	0.284
BMI ≥ 25 kg/m²	1.92 (1.03–3.59)	0.039	

*Abbreviations*: *OR* odds ratio, *CI* confidence interval, *BMI* body mass index

## Discussion

The relationship between periodontitis and cardiovascular disease has been well documented in numerous studies, although some findings have been less consistent. Several recent meta-analyses have confirmed a significant association between periodontal disease and CVD [23, 24], while others [25, 26] have emphasized residual heterogeneity and the possibility of attenuation after extensive confounder adjustment. In this retrospective study involving 472 patients over a two-year period, we found that periodontitis was significantly associated with CVD even after adjustment for established risk factors such as age, sex, smoking, diabetes, and hypertension. Patients with periodontitis had a higher overall prevalence of CVD and higher rates of coronary artery disease and myocardial infarction. Importantly, logistic regression analyses showed that periodontitis remained independently associated with CVD; however, given the retrospective observational design, these findings should be interpreted as associative rather than causal.

Our findings are consistent with prior epidemiological research, which has increasingly highlighted a link between oral inflammatory diseases and cardiovascular outcomes. For example, large-scale cohort studies from Sweden and Korea have demonstrated that patients with severe periodontitis have significantly higher risks of myocardial infarction and stroke, with hazard ratios ranging from 1.2 to 2.0 [17, 19]. Meta-analyses have further supported these associations [13, 18, 27], suggesting that periodontal disease increases the likelihood of cardiovascular morbidity and mortality. Regional variations in oral hygiene practices, socioeconomic factors, and access to preventive dental care may partially account for differences in periodontitis prevalence observed across studies. In our cohort, the prevalence of periodontitis (61%) among patients with a mean age of 59 years closely aligns with epidemiological data reported in Chinese populations, such as those described by Cao et al. [28], supporting the representativeness of our findings. The recent consensus report by Herrera et al. [29] emphasized that periodontal diseases are associated with cardiovascular diseases, diabetes, and respiratory diseases and highlighted the importance of considering periodontal status within broader systemic health assessment. In addition, Van Dyke et al. [30] reported that inflammation of the periodontium was associated with an increased risk of future cardiovascular events. Against this background, the adjusted odds ratio observed in our study (OR = 1.87) is broadly consistent with findings reported in European and North American populations, while also extending this body of evidence to a regional Chinese clinical cohort. The present study therefore adds region-specific evidence from a population in which relatively fewer retrospective cohorts have been published.

Cardiovascular disease comprises a heterogeneous spectrum of clinical entities with distinct pathophysiological mechanisms. In the present study, although a composite CVD outcome was used as the primary endpoint, disease-specific analyses suggest that the observed association with periodontitis was mainly driven by atherosclerotic outcomes, particularly coronary artery disease and myocardial infarction. This pattern is biologically plausible, as chronic periodontal inflammation has been more consistently linked to endothelial dysfunction, lipid metabolism abnormalities, and atherogenesis than to non-atherosclerotic mechanisms underlying heart failure or certain types of stroke. The weaker associations observed for stroke and heart failure may reflect heterogeneity in etiology, limited statistical power, or competing risk factors not directly related to periodontal inflammation.

Nevertheless, not all studies have reached consistent conclusions regarding the association between periodontitis and cardiovascular disease. Reviews of the literature have noted that some prospective studies reported weaker or non-significant associations after more extensive adjustment for socioeconomic and behavioral confounders [25, 26]. Such discrepancies may reflect heterogeneity in study design, periodontal diagnostic criteria, cardiovascular outcome definitions, and the extent of confounder adjustment. In the present study, we sought to minimize these sources of bias by applying standardized periodontal definitions based on the 2017 World Workshop classification and by evaluating a broad spectrum of cardiovascular pathologies rather than a single composite outcome. Although the present study did not directly measure circulating inflammatory cytokines or molecular biomarkers, the observed association between periodontitis and cardiovascular disease may be partially explained by systemic inflammatory mechanisms proposed in previous studies. Periodontitis is characterized by a dysbiotic biofilm and a sustained host immune response, which has been associated with chronic inflammation and systemic dissemination of bacteria and their byproducts in experimental and clinical research [31]. Previous studies have reported the presence of periodontal pathogens, such as Porphyromonas gingivalis and Aggregatibacter actinomycetemcomitans, within atheromatous plaques, suggesting a potential link between periodontal infection and vascular injury [32]. Inflammatory mediators released during periodontal inflammation, including C-reactive protein, interleukin-6, and tumor necrosis factor-α, have been implicated in endothelial dysfunction and atherogenesis in prior literature [31, 32]. However, these mechanistic pathways were not directly assessed in the current analysis and should therefore be interpreted as supportive background evidence rather than study-derived conclusions. Accordingly, our findings primarily highlight clinical associations at the disease-phenotype level rather than providing direct mechanistic insight.

Another pathway involves altered hemostasis and coagulation. Periodontitis has been associated with elevated platelet activation and fibrinogen levels, which may predispose to thrombus formation and acute coronary syndromes [33]. Additionally, dyslipidemia induced by periodontal inflammation may further compound cardiovascular risk. Taken together, these mechanisms provide a biologically plausible rationale for the clinical association observed in our study, although they do not establish causality. Recent evidence further supports the mechanistic link between periodontal inflammation and vascular dysfunction. Multiple meta-analyses have consistently demonstrated that periodontal therapy, particularly non-surgical approaches such as scaling and root planing, significantly improves endothelial function as assessed by flow-mediated dilation (FMD) [34–38]. These studies collectively indicate that reducing periodontal inflammation leads to lower circulating levels of C-reactive protein and interleukin-6, enhanced nitric oxide bioavailability, and partial recovery of endothelial responsiveness. The improvement in vascular function following periodontal treatment suggests that the systemic effects of chronic oral inflammation are, to some extent, reversible. Furthermore, these findings emphasize that endothelial dysfunction may represent a key intermediate mechanism linking periodontitis to atherosclerotic disease progression. Integrating our present results with these meta-analytic findings highlights the biological plausibility of the association observed in our cohort and underscores the importance of maintaining periodontal health as part of comprehensive cardiovascular risk management. An important finding of this study is the differential distribution of cardiovascular pathologies among patients with periodontitis. Coronary artery disease and myocardial infarction were more prevalent in the periodontitis group, whereas associations with stroke and heart failure were comparatively weaker. This pattern suggests that periodontal inflammation may be more strongly linked to atherosclerotic and ischemic cardiovascular processes rather than to non-atherosclerotic or hemodynamic cardiac conditions. These observations underscore the importance of examining disease-specific cardiovascular outcomes rather than relying solely on composite cardiovascular endpoints when evaluating the systemic impact of periodontitis.

From a clinical standpoint, our findings highlight the importance of integrating oral health into systemic disease prevention frameworks. Current cardiovascular risk assessment largely focuses on traditional factors such as hypertension, diabetes, and smoking. However, periodontal status is seldom incorporated into routine cardiovascular evaluation. The severity-stratified findings in our study suggest that advanced or rapidly progressive periodontitis may identify a subgroup with higher cardiovascular vulnerability. By contrast, the subgroup analyses by age, sex, smoking status, and BMI showed generally consistent associations, and the interaction tests were not statistically significant; these subgroup differences should therefore be interpreted cautiously. Early identification and management of periodontitis may serve as a practical adjunct to conventional cardiovascular prevention strategies. A key strength of the present study is its focus on a Chinese clinical population, which has been underrepresented in previous epidemiological studies on the association between periodontitis and cardiovascular disease. Differences in genetic background, lifestyle factors, oral hygiene practices, and healthcare access may influence both periodontal disease patterns and cardiovascular risk profiles. Our findings provide region-specific evidence supporting the relevance of periodontal health in cardiovascular risk stratification within Chinese populations and help address an important gap in the existing literature.

An important extension of the present study is the additional stratified analysis according to periodontal stage and grade, which showed that the association between periodontitis and cardiovascular disease was more pronounced in patients with stage III/IV and grade C disease than in those with milder categories. This finding supports the concept that not all periodontitis carries the same systemic relevance and that more advanced or rapidly progressive disease may confer a greater cardiovascular burden. Such a pattern is biologically plausible, as severe periodontitis is characterized by deeper periodontal pockets, greater clinical attachment loss, more extensive alveolar bone destruction, and a larger ulcerated epithelial surface through which periodontal pathogens, endotoxins, and inflammatory mediators may enter the systemic circulation. In parallel, grade C periodontitis reflects a more rapidly progressive phenotype that may be associated with heightened host inflammatory response and greater microbial challenge. Together, these features may amplify systemic inflammation and endothelial dysfunction, thereby strengthening the observed association with atherosclerotic cardiovascular disease. This interpretation is also consistent with previous severity-based periodontal research, including reports linking periodontitis with worse COVID-19 outcomes and higher systemic inflammatory biomarker levels, suggesting that advanced disease may be accompanied by a greater systemic inflammatory burden than earlier stages [39, 40]. Although our study does not establish causality, these findings reinforce the clinical relevance of incorporating periodontal severity into cardiovascular risk interpretation. More broadly, they also highlight the importance of improving awareness of periodontal health among physicians, particularly cardiology-related clinicians, so that oral health can be more appropriately integrated into patient education, risk-factor assessment, and multidisciplinary preventive care.

At the public health level, the results emphasize the need for greater collaboration between dental and medical disciplines. Interdisciplinary approaches that include routine oral screening in cardiology clinics, and cardiovascular risk counseling in dental settings, may improve patient outcomes. Given the high prevalence of periodontitis in the adult population, even modest reductions in cardiovascular risk attributable to periodontal therapy could translate into significant reductions in disease burden at the population level.

Several limitations should also be acknowledged. First, although adjustment was performed for multiple established cardiovascular risk factors, residual confounding cannot be excluded. Information on medication use (including statins, antihypertensive agents, and antidiabetic drugs), detailed socioeconomic indicators beyond education level, and oral health–related behaviors such as dental attendance, oral hygiene practices, and prior periodontal treatment was not consistently available due to the retrospective nature of the dataset. These unmeasured factors may have influenced both periodontal status and cardiovascular outcomes and could partially explain the observed associations. Second, socioeconomic and lifestyle factors such as diet, physical activity, and access to healthcare were not available, though these variables may significantly influence both oral and cardiovascular outcomes. Third, while our analysis adjusted for major comorbidities, we did not evaluate the effect of periodontal treatment or oral hygiene practices, which could provide important insights into whether improving periodontal health mitigates cardiovascular risk. Finally, the single-center nature of this study may limit generalizability, underscoring the need for multicenter and prospective investigations.

Future research should focus on prospective, multicenter studies with longer follow-up to validate these findings. Stratifying patients according to periodontitis severity and treatment history may clarify dose–response relationships and treatment effects. Interventional trials testing whether periodontal therapy reduces cardiovascular events would provide the most definitive evidence of causality. Additionally, incorporating molecular and imaging biomarkers, as well as machine learning models, could improve risk prediction and facilitate precision medicine approaches to managing both oral and systemic health.

In summary, while this study contributes valuable regional data supporting the periodontitis–CVD link, its retrospective design precludes causal inference. The results should therefore be interpreted as associative rather than deterministic. Future prospective multicenter studies incorporating inflammatory biomarkers, microbial profiling, and longitudinal follow-up are warranted to clarify causality and underlying mechanisms.

## Conclusion

In conclusion, periodontitis was independently associated with a higher likelihood of cardiovascular disease after adjustment for traditional risk factors. These findings support the relevance of periodontal health in comprehensive cardiovascular risk assessment, while prospective studies are needed to clarify causality and the potential benefit of periodontal intervention.

## Supplementary Information

Supplementary Material 1.

## Data Availability

The datasets used and/or analysed during the current study are not publicly available due to patient privacy considerations but are available from the corresponding author on reasonable request.

## Abbreviations

AAP: American Academy of Periodontology
BMI: Body mass index
BOP: Bleeding on probing
CAL: Clinical attachment loss
CI: Confidence interval
CVD: Cardiovascular disease
EFP: European Federation of Periodontology
FMD: Flow-mediated dilation
HbA1c: Glycated hemoglobin
ICC: Intraclass correlation coefficient
IQR: Interquartile range
LDL: Low-density lipoprotein
OR: Odds ratio
PD: Probing depth
PI: Plaque index
PPD: Probing pocket depth
SD: Standard deviation
STROBE: Strengthening the Reporting of Observational Studies in Epidemiology
Tregs: Regulatory T cells
