# Supplementary material for: Association between periodontitis and cardiovascular disease: a retrospective analysis
Source: BMC Oral Health. 2026 Apr 6;26:955. doi: 10.1186/s12903-026-08282-x (PMC13235136; doi:10.1186/s12903-026-08282-x)
Supplement: Supplementary file 1 — Supplementary Material 1. [file 12903_2026_8282_MOESM1_ESM.docx]

Supplementary Table 1. Association of cardiovascular disease with stage- and grade-based severity of periodontitis

| Severity category | Total, n | CVD present, n (%) | CVD absent, n (%) | Unadjusted OR (95% CI) | P value | Adjusted OR (95% CI)* | P value |
| --- | --- | --- | --- | --- | --- | --- | --- |
| **By periodontal stage** |  |  |  |  |  |  |  |
| No periodontitis | 183 | 39 (21.3) | 144 (78.7) | Reference | — | Reference | — |
| Stage I/II periodontitis | 118 | 33 (28.0) | 85 (72.0) | 1.43 (0.84–2.45) | 0.186 | 1.32 (0.76–2.29) | 0.317 |
| Stage III/IV periodontitis | 171 | 79 (46.2) | 92 (53.8) | 3.17 (1.99–5.04) | <0.001 | 2.41 (1.48–3.91) | <0.001 |
| **By periodontal grade** |  |  |  |  |  |  |  |
| No periodontitis | 183 | 39 (21.3) | 144 (78.7) | Reference | — | Reference | — |
| Grade A/B periodontitis | 149 | 48 (32.2) | 101 (67.8) | 1.75 (1.07–2.87) | 0.026 | 1.49 (0.89–2.49) | 0.129 |
| Grade C periodontitis | 140 | 64 (45.7) | 76 (54.3) | 3.11 (1.91–5.05) | <0.001 | 2.28 (1.37–3.79) | 0.002 |

**Abbreviations:** CVD, cardiovascular disease; OR, odds ratio; CI, confidence interval.
*Adjusted for age, sex, smoking history, body mass index, diabetes mellitus, and hypertension.
